# A systematic review of ethnic minority women’s experiences of perinatal mental health conditions and services in Europe

**DOI:** 10.1371/journal.pone.0210587

**Published:** 2019-01-29

**Authors:** Helen Watson, Deborah Harrop, Elizabeth Walton, Andy Young, Hora Soltani

**Affiliations:** 1 Faculty of Health and Wellbeing, Sheffield Hallam University, Sheffield, South Yorkshire, United Kingdom; 2 Academic Unit of Primary Medical Care, The University of Sheffield, Sheffield, South Yorkshire, United Kingdom; Erasmus Medical Center, NETHERLANDS

## Abstract

**Background:**

Women from ethnic minority groups are at greater risk of developing mental health problems. Poor perinatal mental health impacts on maternal morbidity and mortality and can have a devastating impact on child and family wellbeing. It is important to ensure that services are designed to meet the unique needs of women from diverse backgrounds.

**Aim:**

The aim of the review was to explore ethnic minority women's experiences of perinatal mental ill health, help-seeking and perinatal mental health services in Europe.

**Data sources:**

Searches included CINAHL, Maternity and Infant Care, MEDLINE and PsycINFO with no language or date restrictions. Additional literature was identified by searching reference lists of relevant studies.

**Design:**

This was a mixed method systematic review. Study selection, appraisal and data extraction were conducted by two researchers independently. A convergent approach was adopted for the analysis and the data were synthesised thematically.

**Results:**

The 15 eligible studies included women from a range of minority ethnic backgrounds and were all undertaken in the United Kingdom (UK). Seven overarching themes were identified; awareness and beliefs about mental health, isolation and seeking support, influence of culture, symptoms and coping strategies, accessing mental health services, experiences of mental health services and what women want.

**Conclusion:**

Lack of awareness about mental ill health, cultural expectations, ongoing stigma, culturally insensitive and fragmented health services and interactions with culturally incompetent and dismissive health providers all impact on ethnic minority women's ability to receive adequate perinatal mental health support in the UK. Future research should focus on in-depth exploration of the experiences of these women across multiple European settings and interventions to reduce health inequalities among vulnerable mothers and families affected by perinatal mental ill health.

## Introduction

Mental ill health is an urgent European public health and socioeconomic challenge [1]. Mental health disorders are among the most common morbidities of the perinatal period [2], and although their incidence is reported to vary in different settings, approximately 10% of childbearing women are affected during pregnancy and 13% of women after birth [3]. Mental health is integral to safe motherhood, and should be included in all initiatives, programmes, and recommendations for standard care [4].

Mental health disorders may pre-exist or may develop during the perinatal period, and include; depression, anxiety, post-traumatic stress disorder (PTSD), eating disorders, personality disorders, bipolar disorder, affective psychosis and schizophrenia [5,6]. Perinatal mental ill health is a significant risk factor for maternal mortality. Recent evidence from the United Kingdom (UK) suggest that 23% of women who died in the postnatal period suffered from mental health disorders, and suicide is now the second leading cause of maternal death in the UK [7]. Adverse consequences of perinatal mental health disorders are not limited to mothers. They can also result in poor pregnancy outcomes including preterm birth and low birthweight, and poor maternal-infant interaction which is associated with child behavioural, emotional and cognitive problems [8]. Maternal mental ill health has a devastating impact on children's and families' lives [9–12], as well as resulting in considerable cost and resource implications for health services [1,11].

In the UK, ethnic minority groups have a higher burden of common mental health disorders when compared to the majority white population [12, 13, 14, 15] and it is less likely that these will be detected or treated [12,16] including during the perinatal period [12]. Whilst the reasons for these disparities are not fully explained, minority ethnicity may be associated with exposure to psychosocial triggers such as deprivation and social isolation [17,18], discrimination [19], being a migrant, refugee or asylum seeker [15,20], and inequity in health care access and support [12,13,21]. It is important to recognise that ‘minority ethnicity’ is not a homogenous group but encompasses diverse groups of individuals with varied levels of exposure to the aforementioned risks. However, it is also important to acknowledge that there is a complex relationship between the burden of disease and socioeconomic status (SES), particularly as there is wide evidence of non-equivalence of SES across racial groups and the ongoing impact of interpersonal and institutional discrimination [22].

It is of paramount importance to explore the experiences of women from minority ethnic backgrounds, of perinatal mental health, help-seeking and the use of mental health services to inform the development of European maternal mental health services and future interventions to address the perinatal mental health needs of women from minority ethnic groups. To our knowledge there has been no previous systematic review of the evidence of experiences of perinatal mental health and its related services from the perspectives of women from minority ethnic backgrounds who live in Europe. Our review therefore aimed to address the following questions:

1. What are the experiences and perceptions of perinatal mental health amongst ethnic minority women in Europe?
2. What are the experiences and perceptions of perinatal mental health services amongst ethnic minority women in Europe?
3. What factors influence the help-seeking behaviours of ethnic minority women in Europe in relation to perinatal mental health disorders?

## Methods

The methodology of this review has been described in detail in a previously published protocol through PROSPERO (CRD42017077281). This review is reported in line with the PRISMA guidelines [23] (See Supporting Information S1 File). The systematic review applied the principles of mixed-methods research to integrate results from qualitative, quantitative and mixed-methods studies [24], based on the rationale that the retrieval of qualitative and quantitative data within a review can maximise the usefulness of the synthesis by providing an understanding of human experience alongside empirical evidence about a particular phenomenon [25].

### Search strategy

Four databases were searched; CINAHL (EBSCO), Maternity and Infant Care (Ovid), MEDLINE (EBSCO), and PsycINFO (ProQuest). The searches in MEDLINE and CINAHL were undertaken in August 2017, PsycINFO in Sept 2017, and Maternity and Infant Care in October 2017. The search strategy comprised four facets with terms relating to: (1) the perinatal period, (2) ethnicity or possible countries of origin (3) mental health, and (4) research undertaken in Europe. All terms were searched for in the title and abstract fields and, in addition, the fourth facet was searched for in the country of publication field or the nearest equivalent. Controlled vocabulary terms were used where available. The Boolean operators AND and OR were used, alongside truncation, phrase searching and proximity operators. The search syntax was adapted for each database. The full search strategy, as applied in MEDLINE (EBSCO interface) is provided in Supporting Information S2 File.

### Inclusion criteria

Studies were eligible for inclusion if they were primary qualitative, quantitative or mixed methods studies. Studies must have explored perinatal mental health disorders, help-seeking or use of mental health services in women from ethnic minority populations living in Europe. Any interventions targeting this group were eligible for inclusion provided the paper reported self-reported experiences, preferences or health-seeking behaviours associated with perinatal mental health from ethnic minority women's perspectives. Studies were not excluded on the basis of language or publication date.

### Study selection

All papers were screened by title and abstract to determine their eligibility for inclusion in the review. The full-texts of all remaining papers were screened to determine their inclusion/exclusion from the review. Ten percent of papers were independently double checked by a second reviewer and discrepancies in judgement regarding eligibility discussed and resolved within the research team. Reference lists of included papers were checked for further relevant papers.

### Quality assessment

The quality of each paper was assessed, however, no papers were excluded on the basis of low quality scores as there is no empirical evidence to justify this approach [26]. A total of 10% of the articles were appraised by a second reviewer with referral to a third reviewer if disagreement was not resolved. All included qualitative articles were quality appraised using the qualitative National Institute for Health and Care Excellence (NICE) appraisal tool [27]. An overall rating of the quality of the article was classified as high (++) if the majority of the appraisal criteria were met and the study was judged to be trustworthy and reliable and there was significant evidence of author reflexivity. An acceptable quality score (+) required that most criteria were met and identified that there may be some flaws in the study resulting in a lack of rigor. A low-quality score (-) was assigned if either most criteria were not met, or it was judged that there were significant flaws in the study design.

Quality of the included quantitative papers was similarly appraised using the critical appraisal checklist for a questionnaire-based survey study adopted by the National Institute for Health and Clinical Excellence (NICE) [28] for evidence synthesis in guideline development. A high quality score (++) was assigned if the majority of the criteria were met, and it was judged that there was little or no risk of bias. An acceptable quality score (+) required that most criteria were met, and that there may be some flaws in the study with an associated risk of bias. A low quality score (-) was assigned if either most criteria were not met, or it was judged that there were significant flaws relating to key aspects of study design.

### Data extraction

Data extraction was managed in Microsoft Excel using a data extraction proforma developed specifically for this review. Extracted data included author/s, year of publication, study aim, setting, sample size, participant demographics, data collection method, method of analysis and outcomes. Relevant outcome data were extracted from sections of the qualitative studies entitled findings, results, discussion, interpretations or conclusions and were imported directly into NVivo-11 software (QSR International).

### Synthesis

A convergent approach to data synthesis was adopted [29]. This approach was undergirded by the belief that both qualitative and quantitative studies can address the same research questions and produce data that can be readily transformed into each other and that differences between the studies do not warrant separate analyses [30]. In this case quantitative findings were transformed into qualitative form as themes, so that they could be combined with the qualitative findings [29,30] using a thematic synthesis approach [31]. Thematic synthesis included line by line coding, organising codes into descriptive themes and generating analytic themes. The findings from 20% of the papers were independently synthesised by two reviewers in order to ensure a consistent approach.

### Confidence in the findings

The confidence in the findings was assessed using the Confidence in the Evidence from Reviews of Qualitative Research (CERQual) approach [32]. This systematic approach enables the reviewers to produce a score ranging from low to high confidence in the review findings by considering; the methodological limitations of the individual studies, relevance to the review question, coherence and adequacy of the data [32]. The CERQual scores were assessed by two reviewers and then discussed amongst the review team to ensure agreement.

## Findings

**Fig 1:**
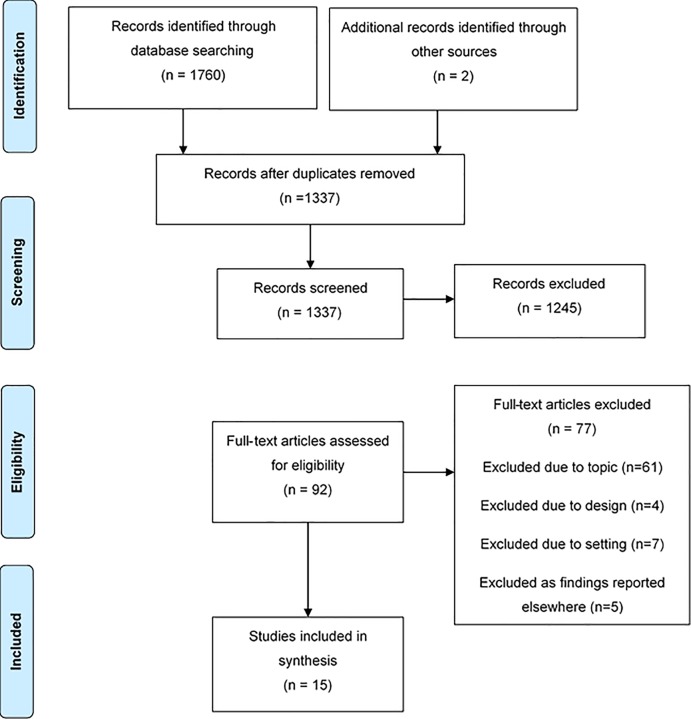
Study selection diagram.

The systematic search identified a total of 1762 references. After the removal of duplicates 1337 papers remained. A total of 92 papers met the criteria for full-text review and 15 were included. A flow diagram of the study search and selection process can be seen in Fig 1, in line with current reporting guidelines [23].

### Characteristics of the included studies

The characteristics of the 15 included studies [33–47] can be seen in Table 1. Twelve of the papers contained qualitative data obtained using interviews [35,36,38,40,41,44,46,47], focus groups [35,37,39,43,46] and observation [33]. Three of the studies collected data using surveys [34,42,45]; all included some narrative summary of their findings, and two presented descriptive statistics [42,45] which were converted to qualitative themes for purpose of this review (See Supporting Information S1 Table). Although the search strategy included all European countries, all of the studies which met the inclusion criteria were undertaken in the UK. The date of publication of the studies ranged from 1984 to 2016, with all but one study [35], published since 2003. The ethnicity or nationality of study participants were categorised as; Bangladeshi [33,39,42,43,46,47], Pakistani [42,47], Indian [42,46,47], South Asian [41], Asian [45], Other Asian [46], Mixed Asian/British [44], Pathan [35], Black [45], Black African [44], Nigerian [38], Ghanaian [38], Black Caribbean [36,37], Portuguese [46], White American [44], White Australian [44], White other [44], other ethnicity [45,46], unspecified black or minority ethnicity [34], and unspecified ethnicity in the case of a group of asylum seekers presumed to be non-native [40]. Although some studies included data from English [33] or White British participants [44,45], this was excluded during data extraction where possible. The studies were of mixed quality, two were judged to be of high quality [45,47], nine of acceptable quality [36–39,41–44,46] and four of low quality [33–35,40]. A summary of the quality scores for each paper are shown in Table 1 with full details in Supporting Information files S2 Table and S3 Table.

**Table 1:** Characteristics of included studies.

Study	Aim	Setting	Participants		Data collection	Data analysis	Quality score
			Sample size	Ethnicity			
Almond [33]	To investigate equity in the provision of a public health nursing postnatal depression service.	England—UK	21	English and Bangladeshi	Observation and interviews	Thematic content analysis	-
Cantle [34]	To identify barriers to partnership working in relation to perinatal mental health in their area, to explore issues and identify solutions to problems and agree to and implement a plan of action.	England—UK	60	Unspecified Black and Ethnic Minority Groups	Survey	Not specified	-
Currer [35]	To explore Pathan women's perpectives on mental health and mental ill health	England—UK	50	Pathan	Interviews and group sessions	Not specified	-
Edge [36]	To explore the models, experiences, and meaning of perinatal depression held by Black Caribbean women	England—UK	12	Black Caribbean	In-depth interviews	Constant comparative approach	+
Edge [37]	To examine Black Caribbean women's perspectives on what might account for low levels of consultation for perinatal depression.	England -UK	42	Black Caribbean	Focus group discussions	Framework analysis	+
Gardner [38]	To explore the lived experience of postnatal depression in West African mothers living in the UK.	England—UK	6	Nigerian and Ghanaian	Semi-structured interviews	Interpretive Phenomenological Analysis	+
Hanley [39]	To explore Bangladeshi mothers' interpretations of postnatal depression and its effect on the wellbeing on the mother, family and community.	Wales—UK	10	Bangladeshi	Focus group interviews	Thematic analysis	+
McLeish [40]	To describe the maternity experiences of asylum seekers in the UK.	England—UK	33	Not specified	Interviews	Not specified	-
Masood [41]	To assess the acceptability and overall experience of the Positive Health Programme by British South Asian mothers.	England—UK	17	South Asian	In-depth interviews	Thematic analysis	+
Noor [42]	To explore the relationship between infant feeding and maternal mental well-being among women of Bangladeshi and Pakistani ethnicity.	England—UK	86	Bangladeshi, Pakistani and Indian	Survey undertaken in structured interviews	Descriptive statistics	+
Parvin [43]	To explore first-generation Bangladeshi women’s understandings and experiences of postnatal distress, and to describe coping strategies during the postnatal period.	England—UK	25	Bangladeshi	Focus groups	Thematic content analysis	+
Raymond [44]	To explore depression during pregnancy amongst women living in an area of socio- economic deprivation.	England—UK	9	Black African, Mixed Asian/British, White American, White Australian, White other, White British	Individual semi-structured interviews	Thematic analysis	+
Redshaw [45]	To find out which women are asked about their mood and mental health during pregnancy and postnatally, and about offer and uptake of treatment.	UK	4571	Mixed, Asian, Black, Other, White	Postal Survey	Descriptive statistics, univariate analysis and logistic regression.	++
Templeton [46]	To describe the experiences of women suffering from postnatal depression in black and minority ethnic communities.	England—UK	18	Bangladeshi, Indian, other Asian, Portuguese, Other	Semi-structured interviews and focus groups	Descriptive thematic analysis	+
Wittkowski [47]	To understand the experience of PND in South Asian mothers living in Great Britain.	England -UK	10	Indian, Pakistani, Bangladeshi	Individual interviews	Constant comparison method	++

## Themes

After in-depth familiarisation and coding the data, seven overarching analytic themes were identified; awareness and beliefs about mental health, influence of culture, symptoms and coping strategies, isolation and seeking support, accessing mental health services, experiences of mental health services and what women want. These themes and their specific sub-themes are described below.

### Awareness and beliefs about mental ill health

Several studies demonstrated that some women from minority ethnic groups were not aware of perinatal mental health disorders [34–36,38,39,42,43,46]. Despite being familiar with or experiencing perinatal mental health symptoms, they didn't consider them to be an illness [36,38,39], didn't have the language to describe the collective symptoms as a disorder [34,39,47], or adopted alternative explanations for the symptoms which included practical problems [43], lack of rest [43], isolation [38] lack of support [38,43], the influence of evil spirits [39] or something that would just go away [46].

*"…don’t know what call it*, *in Pakistan where used to live*, *they don’t have depression there*. *There is no word in Urdu for depression*, *I don’t know what is happening to me*, *never seen it before*, *only when moved here*.*"* South Asia mother ([47], p.486])

*"I’ve had two babies now*, *I don’t know what postnatal depression is supposed to be*, *how you’re supposed to feel*, *look or whatever*, *I don’t know*. *I have no idea*. *What exactly is postnatal depression*? *What are you supposed to be doing*, *saying*, *or whatever*?*"* Black Caribbean mother ([36], p.18-19)

Other women who were able to recognise their symptoms as a mental health disorder identified contributing factors that included financial problems [36], difficulties in personal relationships [36], hormonal factors [36], pregnancy-related physical health problems [36], racist incidents [35], living in a different country [38,47], stress [38], lack of community [38] and issues related to being an asylum-seeker such as the loss of homes, communities, jobs and family members [40].

*"I think it is about the stress……*. *and the [lack of] community*.*"* Black West African mother ([38], p.758)

### Influence of culture

Black Caribbean and South Asian women explained that depression was culturally unacceptable because of its impact on women fulfilling their role in society [36,43]. Women explained that Black Caribbean culture has been influenced by the impact of their history of slavery, and women are expected to be strong and hold the family unit together and are not allowed to have depression as this would be a sign of weakness or not coping [36]. The women reported that they had inherited a cultural legacy and ability to be strong and to cope [36]. Similarly amongst Bangladeshi women, depression was seen as implying weakness [43].

*"I do think that Black people get depression*, *but I don’t think we’re allowed to have depression*. *I think it’s quite a matriarchal society and therefore you’ve got to cope*. *You’ve got to sort your family out*, *and so therefore you are not allowed to be depressed*.*"* Black Caribbean woman ([36], p.19)

Many of the women described that it was culturally unacceptable to talk about problems, feelings or emotional issues to people outside the family or home [36,38,39,43,46], and that if these things were revealed it would result in stigma [38].

*"I didn't just……*. *open up totally…… to them*. *I wouldn't want to… I know it's just the stigma…*. *It's just you know oh… look at the girl…*.. *I think it's just*, *it's just that I don't want the stigma to just keep following me around*.*"* Black West African mother ([38], p.760)

Pathan women explained that as they believed that their health and illness, happiness and unhappiness was all in God's hands, these things should not concern them [35].

### Symptoms and coping strategies

Women who had experience of perinatal mental health disorders described symptoms that included anxiety [40,47], low mood and sadness [40,47], prolonged crying [40], negative thoughts [47], pain in the chest or stomach [46], lethargy [46], behaviour changes such as difficulty leaving the house [47], and appetite and sleep problems [47].

The women responded to their symptoms by minimising their feelings and self-silencing [38,44]. They reported that they adopted coping mechanisms which included; drawing on their inner strength [36,38], solving it themselves [36,43], putting up with it [43], seeking spiritual treatment from a religious leader [39], and other sources of spiritual support such as personal faith [38], private prayer and accepting prayer from others [37,38,43,47].

*"It was my belief and faith in God*, *cause I kept praying*, *my church prayed for me at all times and all that…and I believed that I would be well again*, *it was in my head…so it was… my faith in God*.*"* Black West African mother ([38], p.761)

Women also coped by having time for themselves [47], keeping busy [38] and distracting themselves with activities such as work or shopping or cleaning [38].

*"For me when I am down*, *I just want to do something*, *go out for window shopping or sometimes tidy the house*. *But the best thing for me is to go out*.*"* Black West African mother ([38], p.760)

### Isolation and seeking support

Many women described that they felt emotionally isolated [38,44,47] and that this was exacerbated when they felt misunderstood by their partner, family members or friends [44,47], were physically separated from family members [38,47] or lacked friendships [40,44].

*"My husband just don’t understand how I feel*, *everybody just keep saying Dimaak kharaab hai [mind is not working properly]*.*"* South Asian mother ([47], p.486])

*"The thing is I have nobody*, *I feel totally alone*, *I have no support and this makes me feel worse*.*"* South Asian mother ([47], p.487)

Women described that support from friends [33,38], family [34,38,47], neighbours [46], the spiritual community [36] and work colleagues [46] helped them to cope. Women particularly valued and sought out support from others with the same experiences of poor perinatal mental health in person [38,44,46] or through an online, virtual connection [44].

### Accessing mental health services

Some women were not aware of the support that was available for women with perinatal mental health problems [34,36,38,46,47] and access to support services was influenced by a number of different factors.

#### Women avoiding services

Some women were fearful of accessing support as they felt identification of their symptoms may result in them being judged to be a bad mother or that their children would be removed from them [46]. Others avoided accessing services because they did not want to be labelled with a diagnosis of a mental health problem [40], wanted to avoid taking medication [36,46], or wanted to avoid services which were anticipated to be facilitated by providers who were not of the same ethnic background [35] or were expected to lack compassion based on previous negative experiences [36,37].

*"I wouldn’t wanna particularly unburden myself to some white woman if I’m honest about it*, *it’s about having someone who you can chat to who understands what you’re doing*, *where you’re going*, *where you’re coming from and all that kind of stuff*.*"* Black Caribbean mother ([36], p.22)

*"That is probably why a lot of black women don’t bother going to the system* … *the majority have had nightmares*. *So you’re thinking*, *“What’s the point in going back*?*” It [negative experience during pregnancy and labour] just doesn’t give you any confidence to think they’ll be sympathetic*.*"* Black Caribbean woman ([37], p.258)

#### Overcoming cultural and practical barriers

Women identified that in order to seek support they had to overcome cultural expectations that they would not discuss personal issues outside the family home [36,43]. The identification of their symptoms was limited by their observance of “purdah” whereby they were screened from men and strangers, and hence treated by proxy through their husbands [35] unless treated by a female professional. Language difficulties made it challenging for women to explain their symptoms [36], to ask for support and to access the support that was offered [33,41,46,47].

There were also a number of practical issues that impacted on women accessing perinatal mental health support including; waiting times [36], lack of childcare [41], domestic responsibilities [41], travel costs [41] and the time of day services were provided [41]. Some women found that family members were supportive and facilitated access to support [41], whereas others were restricted from seeking or attending support services by family members [41], or were restricted from expressing their need for services when family members attended appointments with them [39].

#### Attitudes of health care providers

Women found that the attitude of healthcare providers often presented a barrier to accessing support as providers were perceived to be too busy [37,43], didn't ask about perinatal mental health problems [37,43,44,45], were not interested in these problems [43], and failed to recognise the women's symptoms [40,46] or dismissed them [36,47]. Asian and Black women were less likely to be offered treatment than White women [45] and health care providers were perceived to discriminate against the women on account of their ethnicity [36,47].

*"I got answers from professionals like*, *there is nothing wrong with you*, *go back home stop disturbing us*, *basically you are wasting our time*, *and they were horrible* …*I don’t know if they would have said that if I was white*.*"* Pakistani mother ([47], p.487)

#### System issues

Some women described that the model of maternity care that they experienced impacted on access to support for their perinatal mental health problems [44]; women felt more comfortable revealing symptoms to a known carer in a continuity model of maternity care, which contrasted to the compounding feeling of emotional isolation that resulted from a fragmented model of care with different carers at each appointment [44].

*"Every time I went to see the midwife*, *or…*,*I always had somebody different*, *and I don’t want to tell 10 people my story*.*"* White Australian mother ([44], p.45)

### Experiences of perinatal mental health services

#### Lack of culturally appropriate services

Some women found services to be culturally insensitive and felt uncomfortable if they were facilitated by male providers, particularly if their husbands were not attending with them [47].

*"In Pakistan we only saw lady professionals*, *but here you don’t have a choice*, *you have to see the men as well otherwise you don’t get to see a doctor*. *My husband is always at work so he can’t come with me*, *I feel very uncomfortable*.*"* Pakistani mother ([47], p.487)

Women noted that support services were rarely facilitated by professionals from ethnic minority backgrounds [42,46] and that this impacted on their religion and culture not being understood [42]. Women experienced language problems if information was not provided in their first language and interpreters were not available [41]. Where support was facilitated by someone from the same ethnic background, women felt that the sessions were culturally specific and sensitive [41].

*"Because she (the group support facilitator) understood what we go through*, *how our culture is*, *and how our belief systems are*. *She could understand us better than anyone else*.*"* Pakistani mother ([41], p.4)

#### Support groups

Support groups were generally found to be helpful [41,46], and were a safe space for women to share their feelings and be listened to and a way to overcome loneliness [41]. Some women attending group support sessions for women from similar ethnic backgrounds were fearful of breaches of confidentiality and didn't want to talk [41], whereas others felt that group discussions gave them confidence and helped them to overcome anxieties and enabled them to develop strategies, skills and techniques to deal with their mental health problems [41]. Some women in mixed ethnicity groups felt that they didn't fit in or feel part of the group when it was dominated women from a different ethnic background [46].

### What women want

Women identified a number of ways that perinatal mental health service provision for women from ethnic minority backgrounds could be improved. They highlighted that services need to address the full range of mental ill health during the perinatal period [37] and that better multi-agency working was needed [34,37], particularly between the health service and voluntary organisations [34]. They suggested that health professionals would benefit from cultural competency training [41], and that culturally specific support should be provided [47]. Women wanted the opportunity to have early assessment and intervention, and to be provided with information about perinatal mental health problems and services that were available [47]. Women wanted interventions that were regular [44], designed around women's individual preferences [37] and which could be flexible in terms of length, as they felt that some problems required support for longer periods of time than was currently offered [41]. Women also identified that they wanted the opportunity to meet other mothers in similar situations either in group settings [44,47] or in community-based peer support schemes [37,44].

### Confidence in the review findings

A summary of the CERQual scores can be seen in Table 2 with full details in Supporting Information S4 Table. A CERQual score of high confidence in the review findings was applied to five review findings, a further five findings were judged to score moderately, and a score of low confidence was attributed to two findings where the predominant contributing factor that reduced the confidence scores was concern about adequacy as these were based on a small number of studies.

**Table 2:** CERQual scores.

Analytic theme	Review finding	CERQual score
**Awareness and beliefs about mental ill health**	Women lack awareness of perinatal mental health problems	Moderate
**Influence of culture**	Culture impacts on women's experiences of perinatal mental ill health	High
**Symptoms and coping strategies**	Women have a range of strategies for coping with the symptoms of perinatal mental health problems	High
**Isolation and seeking support**	Women with perinatal mental ill health seek various forms of support	High
	Women with perinatal mental ill health feel isolated	High
**Accessing perinatal mental health services**	Some women with perinatal mental health problems avoid services	Moderate
	Women have to overcome practical and cultural barriers to access support services	Moderate
	Healthcare provider's attitudes impact on women's access to perinatal mental health support	High
	The model of maternity care impacts on women accessing support for perinatal mental ill health	Low
**Experiences of perinatal mental health services**	Perinatal mental health services lack culturally sensitivity	Moderate
	Support groups are generally found to be helpful	Low
**What women want**	Women identified a range of ways to improve the provision of services to women from ethnic minority groups	Moderate

## Discussion

### Summary of the review findings

This review has demonstrated the complex issues surrounding ethnic minority women's experiences of perinatal mental ill health, and experiences of seeking and accessing support. Women from ethnic minority backgrounds represent a diverse population with varied beliefs and levels of awareness of perinatal mental health problems and different cultural expectations about their management. For many women the cultural discourse around mental health and the cultural expectations of the role and identity of women in society leaves women feeling isolated, fearful, stigmatised and suffering in silence. This is compounded by culturally inappropriate and fragmented health care services and culturally incompetent health care providers who demonstrate conscious or unconscious bias and poor attitudes towards women seeking help. The lack of published evidence about these issues from other European countries beyond the UK is concerning, particularly in the context of the serious implications of poor perinatal mental health for women, children and their families [8,12].

### The unique experience of poor perinatal mental health for ethnic minority women

The unique aspects of experiencing perinatal mental ill health for women from minority ethnic groups that are identified in this review are further highlighted below by drawing on wider evidence, including that concerning the experiences of majority, white women who are experiencing poor mental health in the context of becoming a mother in the UK.

#### Fear and uncertainty about their role as a mother

Mental ill health continues to threaten a woman's identity as a mother in society. Evidence from White British women resonates with the findings from minority women in this review, identifying that they experience intense fear of being considered to be an unsuitable parent due to their mental health problems, and that this may result in their child being removed from their care [48–55]. However, this review has demonstrated that the threat of or actual loss of identity for women from some minority ethnic groups who are suffering with mental ill health extends further into other aspects of their identity, for example, where they are expected to continue the expression of matriarchal strength and resilience [36] or uphold conflicting religious beliefs [35].

#### Stigma and prejudice

Whilst minority and majority women report fearing or experiencing stigma if their mental ill health is revealed [38,48,56–58], stigma disproportionately affects people from ethnic minority backgrounds who may encounter "double stigma" or the intersectionality of experiencing prejudice and discrimination in healthcare service settings added to the public and internal stigma of suffering with mental ill health [59]. Discrimination is reported to impact on equitable access to healthcare services including mental health services across the UK [60] and this is corroborated by the accounts of women in these studies [36,47] and with the analyses of routinely collected data [12]. The Equality Act 2010 [61] states that NHS treatment and care, including care for perinatal mental health problems, should be equitable and no person should be discriminated against on the basis of their ethnicity. The "Five year forward view for mental health" report [11] highlighted the ongoing racial inequalities in access to mental health care and experience of this care, and that these inequalities have persisted despite the end of the UK government's 5-year "Delivering Race Equality in mental health care" programme [62] and hence, it is clear that addressing the human rights dimensions of equitable access to perinatal mental health care is of paramount importance.

Cultural competence training has been proposed as a strategy to facilitate the provision of culturally appropriate healthcare [63] and address healthcare disparities and inequalities in healthcare encounters by enhancing cultural knowledge, skills or attitudinal responses [64]. This approach is now encouraged or mandated by several professional guidelines and policies [65–67], however, cultural competence training varies widely across the UK [64], and has suffered from a lack of conceptual clarity, rigorous evaluation and institutional buy-in [67]. Hence it is clear that further research is needed to address the modification of health care provider attitudes and behaviours towards women from ethnic minority groups who present with perinatal mental health disorders.

#### Information needs

Women from minority and majority ethnic backgrounds identify an ongoing need for the provision of information about perinatal mental ill health for themselves, their friends and relatives and their communities across the continuum of childbearing from pre-conception to the postnatal period. This includes the signs and symptoms of mental ill health, availability of support and support types and treatment options, risks and benefits [47,68,69]. However, it is evident from this review that language continues to be a barrier preventing some ethnic minority women accessing this information, hence information materials should be available in different languages and healthcare providers should engage the services of qualified interpreters during consultations and ensure understanding.

#### Support needs and preferences

Women's individual support needs and preferences transcend ethnic boundaries and hence it is vital that they are involved in decisions about their treatment, that there are a range of options available and that care is individualised [2]. It is clear from wider evidence that women of all ethnic backgrounds need support that alleviates the isolation of perinatal mental health disorders and that they value professional and peer support in a variety of settings, including the community, and the opportunity to connect with other women who are experiencing similar issues [70]. However, fragmented maternity care provision and dismissive attitudes of health care providers who are perceived to be too busy impact on women from all backgrounds who are trying to access perinatal mental health support [36,44,47,56,71], and there is consensus amongst women that they are most comfortable and most likely to reveal their experiences of negative emotions in the context of continuous relationships [44,72] with non-judgemental, compassionate health care providers [2].

Healthcare providers should be fully educated and equipped to discuss perinatal mental health and wellbeing, and to identify and care for women with perinatal mental health problems [73,74]. This requires clear referral pathways and a coordinated multidisciplinary team approach to ensure that support is available for women who need it [7,75]. The recent UK national maternity review [76] recommends that all women should have the opportunity to establish a relationship with a known carer across the maternity care pathway within the provision of a continuity-of-care model of maternity service provision in the UK, that there should be significant investment in perinatal mental health services, and that mental health should always be considered as part of a woman's personalised care plan. However, it is naïve to ignore the evidence in this review of the impact of women's cultural backgrounds on their presentation and preferences. It is important that healthcare providers are aware of the varied attitudes of women about mental health in order to appropriately identify women suffering with mental health disorders and provide timely support and treatment.

It is vital that services are shaped to meet women's expressed needs and preferences and that women from ethnic minorities are involved in the design of future research, and the co-production of policies, interventions and services. This is in line with current UK health policy which is committed to making service user involvement an intrinsic component of mental health service commissioning and delivery [77–79].

### Review strengths and limitations

To our knowledge this is the first systematic review of literature focussing on the perinatal mental health experiences of women from ethnic minorities in Europe. This review is strengthened by the adoption of a systematic approach in adherence to the a priori protocol, which included a comprehensive search strategy that did not limit study eligibility by language, date or study type. The search identified a relatively high number of relevant studies; however, as all the eligible studies were set in the UK, which limits our findings and may not represent experiences of ethnic minority women from other parts of Europe. The identified gap in the high quality literature from other European countries in such an important area of research and practice, is however one of the main findings of this review. Independent, dual assessment of data extraction and quality assessment of included studies enhanced trustworthiness of the findings. The involvement of a multidisciplinary study team strengthened the development of the search strategy, the final analytic themes, and the assessment of confidence in the findings.

## Conclusion

There is a complex interplay in the factors that influence ethnic minority women's experiences of perinatal mental ill health and access to support for these problems. Cultural expectations, lack of awareness about mental ill health, ongoing stigma, culturally insensitive and fragmented health services and interactions with culturally incompetent and dismissive health providers all impact on women's ability to receive adequate support, and women are left isolated and suffering in silence. Future research should focus on in-depth exploration of the experiences of these women across other European settings to inform the development of effective interventions with the aim of reducing health inequalities among vulnerable mothers and families affected by perinatal mental ill health.

## Supporting information

S1 File Prisma checklist. (DOC)

S2 File MEDLINE search strategy. (DOCX)

S1 Table Transforming QUANT to QUAL. (DOCX)

S2 Table Qualitative quality appraisal. (DOCX)

S3 Table Quantitative quality appraisal. (DOCX)

S4 Table CERQual scoring. (DOCX)
