# Supplementary material for: A systematic review of ethnic minority women’s experiences of perinatal mental health conditions and services in Europe
Source: PLoS One. 2019 Jan 29;14(1):e0210587. doi: 10.1371/journal.pone.0210587 (PMC6351025; doi:10.1371/journal.pone.0210587)
Supplement: S2 File — (DOCX) [file pone.0210587.s002.docx]

**Supporting Information 2. Search strategy for MEDLINE**

The search has been presented as applied in MEDLINE using the EBSCO interface.

Explanation of search terms used: / = MeSH; asterisk; exp = exploded MeSH term; * = denotes any character; "" = phrase search; N = proximity search; ti = title word; ab = abstract word; af = author affiliation

1. peripartum.ti,ab
2. pre-natal.ti,ab
3. prenatal.ti,ab
4. puerperium.ti,ab
5. postnatal.ti,ab
6. childbirth.ti,ab
7. intrapartum.ti,ab
8. childbearing.ti,ab
9. postpartum.ti,ab
10. perinatal.ti,ab
11. peri-natal.ti,ab
12. antenatal.ti,ab
13. pregnan*.ti,ab
14. trimester*.ti,ab
15. puerperal.ti,ab
16. birth*.ti,ab
17. or/1-16
18. exp.pregnancy/
19. peripartum period/
20. postpartum period/
21. exp.pregnancy trimesters/
22. exp.pregnancy, multiple/
23. perinatal care/
24. or/18-23
25. psychiatr*.ti,ab
26. "adjustment disorder*".ti,ab
27. distress.ti,ab
28. "post traumatic stress".ti,ab
29. "mental health".ti,ab
30. "mental illness".ti,ab
31. "mentally ill".ti,ab
32. "mental ill health".ti,ab
33. depression.ti,ab
34. anxiety.ti,ab
35. bipolar.ti,ab
36. schizophrenia.ti,ab
37. "obsessive compulsive disorder*".ti,ab
38. psychosis.ti,ab
39. psychoses.ti,ab
40. phobi*.ti,ab
41. "mood disorder*".ti,ab
42. "dissociative disorder*".ti,ab
43. "eating disorder*".ti,ab
44. anorexi.ti,ab
45. bulimi*.ti,ab
46. or/25-45
47. mental health/
48. exp.mental health services/
49. community mental health services/
50. exp.mental disorders/
51. depression/
52. anxiety disorders/
53. bipolar disoders/
54. exp.bipolar and related disorders/
55. stress disorders, post traumatic/
56. exp.schizophrenia/
57. exp.mood disorders/
58. exp.obsessive-compulsive disorders/
59. panic disorder/
60. exp.schizophrenia spectrum and other psychotic disorders/
61. exp.personality disorders/
62. feeding and eating disorders/
63. anorexia nervosa/
64. binge-eating disorder/
65. bulimia nervosa/
66. depression,postpartum/
67. puerperal disorders/
68. or/47-67
69. 17 N6 46
70. 24 and 68
71. or/69-70
72. ethnic*.ti,ab
73. minorit*.ti,ab
74. BME.ti,ab
75. race.ti,ab
76. racial.ti,ab
77. caste.ti,ab
78. indian*.ti,ab
79. pakistani*.ti,ab
80. chinese.ti,ab
81. arab*.ti,ab
82. bangladeshi*.ti,ab
83. gypsy.ti,ab
84. gypsies.ti,ab
85. traveller*.ti,ab
86. asian*.ti,ab
87. black.ti,ab
88. african*.ti,ab
89. caribbean.ti,ab
90. "central and eastern european*".ti,ab
91. "central european*".ti,ab
92. "eastern european*".ti,ab
93. iraqi*.ti,ab
94. somali*.ti,ab
95. bosnian*.ti,ab
96. serbian*.ti,ab
97. brazilian*.ti,ab
98. surinamese.ti,ab
99. "north african*".ti,ab
100. "south american*".ti,ab
101. romanian*.ti,ab
102. roma*.ti,ab
103. ex-yugoslavia.ti,ab
104. turkish.ti,ab
105. albanian*.ti,ab
106. russian*.ti,ab
107. ethnic groups/
108. minority groups/
109. traffick*.ti,ab
110. migrant*.ti,ab
111. refugee*.ti,ab
112. immigrant.ti,ab
113. immigration.ti,ab
114. migration.ti,ab
115. foreign*.ti,ab
116. asylum.ti,ab
117. exp.human migration/
118. refugees/
119. or/72-119
120. UK.ti,ab.af
121. "united kingdom".ti,ab.af
122. england.ti,ab.af
123. "northern ireland".ti,ab.af
124. scotland.ti,ab.af
125. wales.ti,ab.af
126. britain.ti,ab.af
127. austria.ti,ab.af
128. belgium.ti,ab.af
129. bulgaria.ti,ab.af
130. croatia.ti,ab.af
131. cyprus.ti,ab.af
132. "czech republic".ti,ab.af
133. denmark.ti,ab.af
134. estonia.ti,ab.af
135. finland.ti,ab.af
136. france.ti,ab.af
137. germany.ti,ab.af
138. greece.ti,ab.af
139. hungary.ti,ab.af
140. ireland.ti,ab.af
141. italy.ti,ab.af
142. latvia.ti,ab.af
143. lithuania.ti,ab.af
144. luxembourg.ti,ab.af
145. malta.ti,ab.af
146. netherlands.ti,ab.af
147. poland.ti,ab.af
148. portugal.ti,ab.af
149. romania.ti,ab.af
150. slovakia.ti,ab.af
151. slovenia.ti,ab.af
152. spain.ti,ab.af
153. sweden.ti,ab.af
154. europe.ti,ab.af
155. european.ti,ab.af
156. albania.ti,ab.af
157. andorra.ti,ab.af
158. azerbaijan.ti,ab.af
159. belarus.ti,ab.af
160. bosnia.ti,ab.af
161. herzegovina.ti,ab.af
162. cyprus.ti,ab.af
163. georgia.ti,ab.af
164. kazakhstan.ti,ab.af
165. kosovo.ti,ab.af
166. liechtenstein.ti,ab.af
167. macedonia.ti,ab.af
168. moldova.ti,ab.af
169. monaco.ti,ab.af
170. montenegro.ti,ab.af
171. norway.ti,ab.af
172. russia.ti,ab.af
173. "san marino".ti,ab.af
174. serbia.ti,ab.af
175. slovenia.ti,ab.af
176. switzerland.ti,ab.af
177. turkey.ti,ab.af
178. ukraine.ti,ab.af
179. "vatican city".ti,ab.af
180. "holy see".ti,ab.af
181. "channel islands".ti,ab.af
182. "isle of man".ti,ab.af
183. "faeroe islands".ti,ab,af
184. gibraltar.ti,ab,af
185. europe/
186. austria/
187. belgium/
188. bulgaria/
189. croatia/
190. czech republic/
191. hungary/
192. poland/
193. romania/
194. slovakia/
195. slovenia
196. france/
197. germany/
198. exp.united kingdom/
199. greece/
200. ireland/
201. italy/
202. luxembourg/
203. netherlands/
204. portugal/
205. spain/
206. denmark/
207. finland/
208. sweden/
209. cyprus/
210. estonia/
211. latvia/
212. lithuania/
213. malta/
214. slovenia/
215. albania/
216. andorra/
217. azerbaijan/
218. republic of belarus/
219. bosnia and herzegovina/
220. cyprus/
221. georgia (republic)/
222. kazakhstan/
223. kosovo/
224. liechtenstein/
225. macdeonia (republic)/
226. moldova/
227. monaco/
228. montenegro/
229. norway/
230. russia/
231. san marino/
232. serbia/
233. slovenia/
234. switzerland/
235. turkey/
236. ukraine/
237. "vatican city"/
238. channel islands/
239. gibraltar/
240. or/120-239
241. 71 and 119 and 240
