# Supplementary material for: A systematic review of ethnic minority women’s experiences of perinatal mental health conditions and services in Europe
Source: PLoS One. 2019 Jan 29;14(1):e0210587. doi: 10.1371/journal.pone.0210587 (PMC6351025; doi:10.1371/journal.pone.0210587)
Supplement: S1 Table — (DOCX) [file pone.0210587.s003.docx]

S1 Table. Transforming quantitative findings to qualitative themes

**Noor (2008)**

| **Quantitative findings** | **Transformation to qualitative** |
| --- | --- |
| When asked whether they had heard of the term ‘postnatal depression’, 65% of the women said they had not. | There is a lack of awareness about perinatal mental ill health amongst many women from ethnic minority backgrounds. |
| Women were asked whether they would talk to anyone if they felt sad or unhappy. Twenty-eight per cent of women said ‘yes’ and the rest (72%) said that they do not discuss their unhappiness with anybody. | Many women don't talk about how their negative emotions. |
| Of those who said they did discuss their unhappiness, 54% discussed it with husbands and 46% with female relatives. | About half of women who did discuss their negative emotions, discussed this with their husband or female relative. |
| When asked where they obtained advice or information during pregnancy, childbirth or postnatally, 38.4% of women gained this from female relatives and 31.4% from health professionals. Similarly, when asked whose advice they followed the most, 54.7% of women said advice from female relatives and 24.4% followed advice from health professionals | Female relatives play an important role in the information women receive and decision-making. |
| In terms of women’s own evaluation, 57% of women said that they mostly or partially understood what health services staff told them and 58% of women thought that their views had been mostly or partially understood by the health professional staff.  Only 14% of women felt that they had access to a health professional who could understand the religion and culture of the mother, while 85% expressed a desire to have access to such a health professional. | Some women feel that their views are misunderstood by health professionals and that they don't understand what health professionals tell them.  Women feel that professionals lack cultural competency.  Women want access to health professionals who are culturally competent. |

**Redshaw (2016)**

| **Quantitative findings** | **Transformation to qualitative** |
| --- | --- |
| Of the 946 women (21 %) who disclosed antenatal mental health problems, 36 % reported being offered treatment (Table 1). In univariate analysis, Asian and Black women were substantially and significantly less likely to report being offered treatment, 20 and 18 % respectively compared to 41 % of White women | Asian and Black women were substantially less likely to report being offered treatment than White women. |
