# Supplementary material for: A systematic review of ethnic minority women’s experiences of perinatal mental health conditions and services in Europe
Source: PLoS One. 2019 Jan 29;14(1):e0210587. doi: 10.1371/journal.pone.0210587 (PMC6351025; doi:10.1371/journal.pone.0210587)
Supplement: S2 Table — (DOCX) [file pone.0210587.s004.docx]

**Supporting Information 3. Qualitative study appraisal summary**

| **APPRAISAL DOMAIN** | Almond (2011) | Currer (1984) | Edge (2005) | Edge (2011) | Gardner (2014) | Hanley (2007) | Masood (2015) | Parvin (2004) |
| --- | --- | --- | --- | --- | --- | --- | --- | --- |
| 1  2 | Appropriate  Clear | Appropriate  Mixed | Appropriate  Clear | Appropriate  Clear | Appropriate  Clear | Appropriate  Clear | Appropriate  Clear | Appropriate  Clear |
| 3 | Not sure | Not sure | Defensible | Defensible | Defensible | Defensible | Defensible | Defensible |
| 4 | Appropriately | Inadequately reported | Appropriately | Appropriately | Appropriately | Inadequately reported | Appropriately | Appropriately |
| 5  6  7 | Not described  Clear  Reliable | Not described  Clear  Not sure | Not described  Clear  Not sure | Not described  Clear  Reliable | Not described  Clear  Reliable | Clearly  Clear  Not sure | Not described  Clear  Reliable | Not described  Clear  Reliable |
| 8  9  10  11  12  13 | Rigorous  Rich  Unreliable  Convincing  Relevant  Adequate | Not reported  Poor  Not reported  Not convincing  Relevant  Inadequate | Rigorous  Rich  Not reported  Convincing  Relevant  Adequate | Rigorous  Rich  Reliable  Convincing  Relevant  Adequate | Rigorous  Rich  Reliable  Convincing  Relevant  Adequate | Rigorous  Rich  Not reported  Convincing  Relevant  Adequate | Rigorous  Rich  Reliable  Convincing  Relevant  Adequate | Not reported  Rich  Not reported  Convincing  Relevant  Adequate |
| 14 | Appropriate | Not reported | Not reported | Appropriate | Appropriate | Appropriate | Appropriate | Not reported |
| **OVERALL ASSESSMENT** | **-** | **-** | **+** | **+** | **+** | **+** | **+** | **+** |

| **APPRAISAL DOMAIN** | Raymond (2009) | Templeton (2013) | Wittkowski (2011) |
| --- | --- | --- | --- |
| 1  2 | Appropriate  Clear | Appropriate  Clear | Appropriate  Clear |
| 3 | Defensible | Defensible | Defensible |
| 4 | Appropriately | Inadequately reported | Appropriately |
| 5  6  7 | Clearly  Clear  Reliable | Not described  Clear  Not sure | Clearly  Clear  Reliable |
| 8  9  10  11  12  13 | Rigorous  Rich  Not reported  Convincing  Relevant  Adequate | Rigorous  Rich  Not reported  Convincing  Relevant  Adequate | Rigorous  Rich  Reliable  Convincing  Relevant  Adequate |
| 14 | Appropriate | Appropriate | Appropriate |
| **OVERALL ASSESSMENT** | **+** | **+** | **++** |

Scoring: An overall rating of the quality of the article was classified as acceptable (+) if questions 1,2,3,11,12,13 were achieved. An overall rating of high quality (++) was assigned if questions 5,6,7,8 and 10 were also achieved, as these questions related to trustworthiness, rigour and reliability. Otherwise article quality was classified as low (-).
