# Supplementary material for: A systematic review of ethnic minority women’s experiences of perinatal mental health conditions and services in Europe
Source: PLoS One. 2019 Jan 29;14(1):e0210587. doi: 10.1371/journal.pone.0210587 (PMC6351025; doi:10.1371/journal.pone.0210587)
Supplement: S3 Table — (DOCX) [file pone.0210587.s005.docx]

**Supporting Information 4. Quantitative study quality appraisal**

| **APPRAISAL CRITERIA** | **Cantle (2010)** | **Noor (2007)** | **Redshaw (2016)** |
| --- | --- | --- | --- |
| Was a questionnaire the most appropriate method? | Yes | Yes | Yes |
| Have claims for validity been made, and are they justified? | None made | Uses a validated tool for mood assessment and discusses limitations | The original survey report has been referenced which gives details regarding validity and reliability |
| Have claims for reliability been made, and are they justified? | None made | No claims have been made |  |
| Are example questions provided? | No | No | No |
| Did the questions make sense, and could the participants in the sample understand them? Were any questions ambiguous or overly complicated? | Can't tell | Can't tell | Can't tell |
| Are details given about the piloting undertaken | No | Piloting not discussed | Yes in original report |
| Was the questionnaire adequately piloted in terms of the method and means of administration, on people who were representative of the study population? | Can't tell | Can't tell | Yes |
| Was the sampling frame for the definitive study sufficiently large and representative? | No details given | Yes | Yes |
| Was the method of distribution and administration reported | Not reported | Yes | Yes |
| Were the response rates reported, including details of participants who were unsuitable for the research or refused to take part? | Not reported | Yes | Yes |
| Have any potential response biases been discussed? | No | No | Yes |
| What sort of analysis was carried out and was this appropriate? | Not reported | ANOVA, t-test and chi2 were used appropriately | Descriptive and binary  logistic regression -appropriate |
| Were all relevant data reported? | Unclear | Yes | Yes |
| Are quantitative results definitive (significant), and are relevant non-significant results also reported? | Unclear | Yes | Yes |
| Have qualitative results been adequately interpreted (e.g. using an explicit theoretical framework), and have any quotes been properly justified and contextualised? | Unclear | None reported | None reported |
| Have the researchers drawn an appropriate link between the data and their conclusions? | Unclear | Yes | Yes |
| Have the findings been placed within the wider body of knowledge in the field (e.g. via a comprehensive literature review), and are any recommendations justified? | Very poorly | Yes | Yes |
| **OVERALL SCORE** | **-** | **+** | **++** |
