# Supplementary material for: A systematic review of ethnic minority women’s experiences of perinatal mental health conditions and services in Europe
Source: PLoS One. 2019 Jan 29;14(1):e0210587. doi: 10.1371/journal.pone.0210587 (PMC6351025; doi:10.1371/journal.pone.0210587)
Supplement: S4 Table — (DOCX) [file pone.0210587.s006.docx]

Supporting Information 6. CERQual scoring

| **Analytic theme** | **Review finding** | **Studies contributing to the review finding** | **Methodological limitations** | **Coherence** | **Adequacy** | **Relevance** | **CERQual assessment of confidence in the evidence** |
| --- | --- | --- | --- | --- | --- | --- | --- |
| **Awareness and beliefs about mental ill health** | Women lack awareness of perinatal mental health problems | 2,3,4,6,7,10,11,14,15 | Minor concerns (2 low quality, 6 acceptable quality and 1 high quality) | Moderate concerns | No or minor concerns | No or minor concerns | **MODERATE** |
| **Influence of culture** | Culture impacts on women's experiences of perinatal mental ill health | 2,3,4,6,7,11,14,15 | Minor concerns (2 low quality, 5 acceptable quality and 1 high quality) | No or minor concerns | No or minor concerns | No or minor concerns | **HIGH** |
| **Symptoms and coping strategies** | Women have a range of strategies for coping with the symptoms of perinatal mental health problems | 5,6,7,11,12,14,15 | No or minor concerns  (6 acceptable quality and 1 high quality) | No or minor concerns | No or minor concerns | No or minor concerns | **HIGH** |
| **Isolation and seeking support** | Women with perinatal mental ill health feel isolated | 6,8,12,14,15 | Minor concerns  (1 low quality, 3 acceptable quality and 1 high quality) | No or minor concerns | Minor concerns | No or minor concerns | **HIGH** |
|  | Women with perinatal mental ill health seek various forms of support | 1,2,4,6,9,10,12,14,15 | Minor concerns  (2 low quality, 6 acceptable quality and 1 high quality) | No or minor concerns | Minor concerns | No or minor concerns | **HIGH** |
| **Accessing perianal mental health services** | Some women with perinatal mental health problems avoid services | 4,5,14 | No or minor concerns  (3 studies of acceptable quality) | Minor concerns | Serious concerns  (3 studies and thin data) | No or minor concerns | **MODERATE** |
|  | Women have to overcome practical and cultural barriers to access support services | 1,3,4,7,9,11,14,15 | Minor concerns  (2 studies low quality, 5 studies acceptable quality and 1 study high quality) | Minor concerns | Moderate concerns | No or minor concerns | **MODERATE** |
|  | Healthcare provider's attitudes impact on women's access to perinatal mental health support | 4,5,11,12,13,14,15 | No or minor concerns  (5 studies of acceptable quality, 2 studies high quality) | Minor concerns | Minor concerns | No or minor concerns | **HIGH** |
|  | The model of maternity care impacts of women accessing support for perinatal mental ill health | 12 | No or minor concerns  (1 study of acceptable quality) | Minor concerns | Serious concerns | No or minor concerns | **LOW** |
| **Experiences of perinatal mental health services** | Perinatal mental health services lack culturally sensitivity | 9,10,14,15 | No or minor concerns  (3 studies of acceptable quality and 1 of high quality) | Moderate concerns | Moderate concerns  (Only data from 4 studies, but rich data) | No or minor concerns | **MODERATE** |
|  | Support groups are generally found to be helpful | 9,14 | Minor concerns  (2 studies of acceptable quality) | Moderate concerns | Serious concerns  (Only 2 studies) | No or minor concerns | **LOW** |
| **What women want** | Women identified a range of ways to improve the provision of services to women from ethnic minority groups | 2,5,9,12,15 | Minor concerns  (1 study poor quality, 3 studies of acceptable quality and 1 high quality) | Minor concerns | Moderate concerns | No or minor concerns | **MODERATE** |
